# Noise exposure and dizziness in middle-aged adults: a nationally representative study

**DOI:** 10.3389/fneur.2026.1818481

**Published:** 2026-05-19

**Authors:** Hyunji Kim, Kyoungwoo Kim

**Affiliations:** 1 Department of Neurology, Seoul National University Bundang Hospital, Seongnam, Republic of Korea; 2 Department of Medicine, Graduate School of Medicine, Ewha Womans University, Seoul, Republic of Korea; 3 Department of Neurology, Ewha Womans University Seoul Hospital, Seoul, Republic of Korea; 4 Department of Family Medicine, Inje University Haeundae Paik Hospital, Busan, Republic of Korea

**Keywords:** dizziness, earphone, noise exposure, normal hearing, occupational noise exposure, population-based

## Abstract

**Background:**

Dizziness is a common and multifactorial neurological symptom whose environmental determinants remain incompletely understood. Although noise exposure is well established as a cause of hearing loss, its potential relationship with dizziness has been insufficiently investigated at the population level.

**Objective:**

To evaluate associations between everyday noise exposure and dizziness in middle-aged adults and to examine whether such associations are more detectable among individuals with normal audiometric hearing.

**Methods:**

We analyzed nationally representative data from adults aged 40–59 years participating in KNHANES. Noise exposure was assessed using workplace noise exposure and earphone use duration in noisy environments. Associations with dizziness were evaluated using survey-weighted logistic regression models under three exposure specifications: (1) categorical exposure, (2) continuous exposure using log-transformed earphone duration, and (3) joint exposure categories defined as combinations of workplace noise exposure (yes vs. no) and earphone use duration (none, ≤120 min/day, >120 min/day). Models were fully adjusted for demographic, behavioral, cardiometabolic, and psychological factors. Parallel analyses were conducted among participants with normal audiometric hearing.

**Results:**

In the overall population, associations under categorical and continuous exposure specifications were modest and not consistently statistically significant. Under the joint exposure specification, participants with both workplace noise exposure and earphone use >120 min per day showed higher odds of dizziness (OR 5.03, 95% CI 1.35–19.05), whereas single-exposure categories showed more modest associations. Associations were more pronounced among adults with normal audiometric hearing, with a significant trend observed across increasing earphone exposure levels.

**Conclusion:**

Noise exposure was associated with dizziness-related symptoms at the population level, with more pronounced patterns observed under joint exposure conditions and in individuals with normal audiometric hearing. Further studies are needed to determine whether this association reflects specific vestibular disorders.

## Introduction

Dizziness is a common and multifactorial symptom encountered across adulthood. Population-based studies have shown that dizziness and vertigo are highly prevalent and often coexist with multiple comorbidities (1–3). In real-world settings, dizziness arises within a complex interplay of vestibular, neurological, cardiovascular, metabolic, and psychological influences (4, 5). Because of this etiologic heterogeneity, isolating the contribution of a single environmental exposure is inherently challenging.

Noise exposure is one of the most pervasive environmental stressors in modern society. Its role in cochlear injury and hearing loss is well established (6–8). However, emerging experimental evidence suggests that intense acoustic stimulation may also activate or stress vestibular structures, including the saccule and vestibular afferents (9–14). Acoustic stimulation has been shown to activate otolith-mediated pathways and vestibular afferents, providing neurophysiological plausibility for a noise–vestibular link. A recent systematic review further supports the plausibility of noise-related vestibular dysfunction (15). Despite this biological plausibility, epidemiologic evidence linking noise exposure to dizziness in community populations remains limited.

From a life-course perspective, noise exposure is rarely a single event but rather a repeated and cumulative experience across adulthood (16). Recurrent acoustic stress may incrementally contribute to sensory vulnerability, even in the absence of overt hearing loss. Because dizziness in later life reflects the accumulation of multiple physiological stressors (17–19), modest exposure-related influences may be difficult to detect in heterogeneous populations.

To reduce background heterogeneity and age-related confounding, we focused on adults aged 40–59 years and specifically examined associations among individuals with normal audiometric hearing.

## Methods

### Study design and participants

This study analyzed data from the 2022–2023 cycle of the Korea National Health and Nutrition Examination Survey (KNHANES), a nationally representative cross-sectional survey conducted using a multistage, stratified, clustered probability sampling design (20). Among participants aged ≥40 years, individuals aged 40–59 years who completed the otolaryngological examination were eligible for inclusion. The otolaryngological examination included standardized questionnaires on dizziness and noise exposure, as well as audiometric assessments. A total of 7,675 participants aged ≥40 years were initially identified, of whom 3,377 were aged 40–59 years. Among these, 3,376 participants completed the otolaryngological examination and were included in the analysis. A complete-case approach was applied, and the analytic sample size varied slightly depending on the availability of specific variables (e.g., audiometric data).

All analyses incorporated sampling weights, primary sampling units, and stratification variables to generate nationally representative estimates.

### Assessment of dizziness

Dizziness was assessed using a standardized questionnaire item asking whether participants had experienced dizziness or balance problems during the past 12 months. Responses were dichotomized as “yes” or “no,” and dizziness was analyzed as a binary outcome variable. Throughout the study, dizziness refers to self-reported symptoms and does not imply a clinical diagnosis of vestibular pathology.

### Assessment of noise exposure

Noise exposure was evaluated using both occupational and recreational indicators.

Occupational noise exposure was defined based on self-reported exposure to loud noise at the workplace that interfered with verbal communication, a commonly used proxy for high-intensity noise exposure as speech communication becomes difficult at noise levels around 80–85 dB(A) (21).

Recreational exposure was assessed by asking participants whether they used earphones in noisy environments such as busses or subways, where elevated background noise (approximately 70–85 dB(A)) may lead to increased listening volumes often exceeding 80–90 dB (A) (22, 23). Participants reporting earphone use were additionally asked to estimate their average daily listening time.

Earphone-use duration was categorized into none, ≤60, 60–120, and >120 min/day to create interpretable exposure strata. This categorization was informed by a dose-based safe-listening framework and used >120 min/day as a pragmatic high-exposure category rather than as a prespecified biological breakpoint (24). To evaluate intensity-related effects, earphone duration was additionally modeled as a continuous variable using a natural logarithmic transformation defined as ln (minutes + 1) to account for right-skewed distribution.

To further evaluate combined exposure patterns, a joint noise exposure variable was constructed as mutually exclusive categories based on combinations of workplace noise exposure (yes vs. no) and earphone use duration (none, ≤120 min per day, >120 min per day).

### Audiometric hearing assessment

Pure-tone audiometry was performed in soundproof booths using calibrated audiometric equipment. Air-conduction thresholds were measured at 0.5, 1, 2, and 4 kHz. The pure-tone average (PTA) was calculated as the mean of these frequencies in both ears (25). Audiometric normal hearing was defined as PTA ≤ 25 dB HL in both ears. To reduce background heterogeneity and enhance detectability of potential exposure-related associations, parallel analyses were conducted among participants classified as having audiometric normal hearing. This restriction was prespecified to examine whether associations could be more clearly observed in a lower-background-risk subgroup.

### Covariates

Multivariable models were adjusted for sociodemographic characteristics (age, sex, household income quintiles, and education level), health behaviors [smoking status categorized by pack-years, alcohol consumption (26), and aerobic physical activity defined as ≥600 metabolic equivalent task-minutes per week (27)], physician-diagnosed cardiometabolic conditions (hypertension, diabetes mellitus, and dyslipidemia), perceived stress (four levels), anxiety severity measured using the Generalized Anxiety Disorder-7 scale (GAD-7) (28), and tinnitus severity.

### Statistical analysis

All analyses accounted for the complex survey design of KNHANES, incorporating sampling weights, stratification, and clustering. Weighted descriptive statistics were presented as means with standard errors for continuous variables and weighted percentages for categorical variables.

Associations between noise exposure and dizziness were evaluated using survey-weighted multivariable logistic regression models under three exposure specifications:

1. Categorical exposure specification, in which workplace noise exposure and ordered categories of earphone duration were entered simultaneously. A test for linear trend was performed by modeling earphone duration as an ordinal variable coded in increasing order of exposure.
2. Continuous exposure specification, in which workplace noise exposure and log-transformed earphone duration [ln(minutes + 1)] were included concurrently.
3. Joint exposure specification, in which mutually exclusive joint noise exposure categories were defined based on combinations of workplace noise exposure (yes vs. no) and earphone use duration (none, ≤120 min per day, >120 min per day).

Identical fully adjusted covariate sets were applied across all exposure specifications to ensure comparability. In addition to models fitted in the overall sample, identical fully adjusted models were estimated within the audiometric normal subgroup to evaluate consistency and detectability of associations under reduced background heterogeneity. Statistical significance was assessed using design-adjusted Wald *F* tests, and two-sided *p*-values <0.05 were considered statistically significant.

All analyses were conducted using the Complex Samples module of IBM SPSS Statistics for Windows, Version 29.0 (IBM Corp., Armonk, NY, United States).

The Korea National Health and Nutrition Examination Survey (KNHANES) data are publicly available and fully de-identified. The study protocol was reviewed and approved by the Institutional Review Board of Haeundae Paik Hospital.

## Results

### Participant characteristics

Among 3,376 participants, representing approximately 14.4 million Korean adults aged 40–59 years, the overall weighted prevalence of dizziness was 30.3% (Table 1). Dizziness prevalence was higher among women.

**Table 1:** Sociodemographic, clinical, auditory, and noise exposure characteristics of participants according to dizziness status.

Characteristics	Total	Dizziness	No dizziness	*p*-value
Weighted number	14,381,845	4,363,614	10,018,231	
(Non-weighted)	3,376	1,072	2,304	
Weighted % (SE)	100.0	30.3 (1.0)	69.7 (1.0)	
Demographics				
Sex				
Male	49.4 (0.8)	12.2 (0.7)	37.2 (0.9)	<0.001
Female	50.6 (0.8)	18.1 (0.7)	32.5 (0.8)	
Age, years				
Mean ± SE	49.7 ± 0.15	49.8 ± 0.21	49.6 ± 0.17	0.412
Age group, years				
40–49	48.4 (1.2)	14.9 (0.7)	33.5 (1.1)	0.408
50–59	51.6 (1.2)	15.4 (0.8)	36.2 (1.1)	
Socioeconomic status				
Household income (quintiles)				
1st (lowest)	5.2 (0.5)	2.1 (0.3)	3.1 (0.4)	0.005
2nd	12.7 (0.8)	4.5 (0.4)	8.3 (0.6)	
3rd	22.0 (1.0)	6.8 (0.5)	15.2 (0.8)	
4th	28.0 (1.1)	7.9 (0.5)	20.1 (1.0)	
5th (highest)	32.1 (1.3)	9.1 (0.7)	23.0 (1.1)	
Years of schooling				
≤6 years	2.6 (0.3)	0.8 (0.2)	1.8 (0.3)	0.397
7–12 years	43.3 (1.4)	13.7 (0.8)	29.6 (1.1)	
>12 years	54.1 (1.5)	15.8 (0.8)	38.3 (1.4)	
Health behaviors				
Smoking status				
Never smoker/<5 pack-years	55.7 (1.0)	18.0 (0.7)	37.7 (0.9)	0.029
Former smoker, ≤10 pack-years	12.6 (0.6)	3.5 (0.4)	9.1 (0.6)	
Former smoker, >10 pack-years	10.9 (0.6)	3.3 (0.4)	7.6 (0.6)	
Current smoker, ≤25 pack-years	12.8 (0.7)	3.0 (0.3)	9.8 (0.6)	
Current smoker, >25 pack-years	8.0 (0.6)	2.6 (0.4)	5.4 (0.5)	
Alcohol consumption				
None or <1 time/month	21.1 (0.8)	7.8 (0.5)	13.3 (0.7)	0.390
≥1 time/month without binge drinking	54.5 (1.1)	16.5 (0.7)	37.9 (1.1)	
≥1 time/week with binge drinking	24.4 (1.0)	6.0 (0.5)	18.4 (0.9)	
Meeting aerobic physical activity guidelines				
Yes	48.0 (1.1)	14.1 (0.7)	33.9 (1.0)	0.281
Clinical comorbidities				
Hypertension (physician-diagnosed)	19.1 (0.9)	6.5 (0.6)	12.6 (0.7)	0.089
Diabetes mellitus (physician-diagnosed)	8.9 (0.6)	3.2 (0.3)	5.7 (0.5)	0.040
Dyslipidemia (physician-diagnosed)	21.9 (0.8)	7.4 (0.5)	14.5 (0.7)	0.036
Tinnitus				
Tinnitus severity (self-reported)				
None	92.5 (0.5)	26.9 (0.9)	65.5 (1.0)	<0.001
Mild	2.4 (0.3)	0.8 (0.2)	1.5 (0.3)	
Moderate	3.1 (0.3)	1.3 (0.2)	1.7 (0.3)	
Severe	2.1 (0.3)	1.2 (0.2)	0.8 (0.2)	
Tinnitus chronicity				
None	92.5 (0.5)	26.9 (0.9)	65.5 (1.0)	<0.001
<6 months	2.1 (0.3)	1.1 (0.2)	0.9 (0.2)	
≥6 months	5.4 (0.4)	2.2 (0.3)	3.2 (0.4)	
Psychological factors				
Anxiety severity (GAD-7 score)				
Minimal (0–4)	83.2 (0.7)	22.5 (0.9)	60.7 (1.0)	<0.001
Mild (5–9)	12.8 (0.7)	5.5 (0.5)	7.3 (0.5)	
Moderate (10–14)	2.6 (0.3)	1.4 (0.2)	1.3 (0.2)	
Severe (15–21)	1.3 (0.2)	0.9 (0.2)	0.5 (0.1)	
Perceived stress				
Almost none	12.5 (0.7)	2.7 (0.3)	9.9 (0.6)	<0.001
A little	60.3 (1.0)	16.4 (0.7)	43.9 (1.0)	
A lot	22.7 (0.9)	9.0 (0.7)	13.7 (0.6)	
Very much	4.5 (0.4)	2.2 (0.3)	2.2 (0.3)	
Noise exposure				
Earphone use in noisy environments (min/day)				
None	91.3 (0.6)	27.4 (0.9)	63.9 (1.1)	0.190
≤60 min/day	5.9 (0.5)	2.0 (0.2)	3.9 (0.4)	
61–120 min/day	1.5 (0.3)	0.4 (0.1)	1.1 (0.2)	
>120 min/day	1.3 (0.2)	0.6 (0.2)	0.7 (0.2)	
Workplace noise exposure (yes/no)				
Yes	16.4 (0.8)	5.2 (0.4)	11.2 (0.7)	0.429
Joint noise exposure categories				
No noise exposure	76.6 (1.0)	22.8 (0.8)	53.9 (1.2)	0.298
Earphone ≤120 min/day only	6.1 (0.5)	2.0 (0.3)	4.1 (0.4)	
Earphone > 120 min/day only	0.9 (0.2)	0.3 (0.1)	0.6 (0.1)	
Workplace noise exposure only	14.7 (0.8)	4.6 (0.4)	10.1 (0.7)	
Workplace noise exposure + earphone ≤120 min/day	1.4 (0.2)	0.4 (0.1)	1.0 (0.2)	
Workplace noise exposure + earphone >120 min/day	0.4 (0.1)	0.2 (0.1)	0.1 (0.1)	
Auditory symptoms (self-reported)				
Perceived hearing difficulty (self-reported)				
No perceived hearing difficulty	90.6 (0.7)	26.8 (0.9)	63.9 (1.1)	0.110
Mild perceived hearing difficulty	8.5 (0.6)	3.3 (0.3)	5.3 (0.5)	
Moderate perceived hearing difficulty	0.8 (0.2)	0.3 (0.1)	0.5 (0.1)	
Unable to hear at all	0.0	0.0	0.0	
Audiometric hearing status (both ears)				
Normal hearing (≤25 dB HL, both-ear criterion)	80.1 (0.9)	24.3 (0.9)	55.7 (1.1)	0.836
Mild hearing loss (26–40 dB HL)	10.8 (0.6)	3.3 (0.3)	7.5 (0.6)	
Moderate-or-worse hearing loss (>40 dB HL)	4.0 (0.4)	1.3 (0.2)	2.7 (0.3)	
Not classifiable	5.1 (0.6)	1.4 (0.3)	3.7 (0.5)	

Values are presented as weighted percentages (standard errors) unless otherwise indicated. Age is presented as weighted mean ± standard error. All estimates account for the complex survey design of the Korea National Health and Nutrition Examination Survey (KNHANES). *p*-values were obtained using design-adjusted Wald *F* tests for continuous variables and Rao–Scott adjusted chi-square tests for categorical variables. Dizziness was defined as self-reported dizziness or balance problems experienced during the past 12 months. Subjective hearing difficulty reflects self-perceived hearing status assessed by questionnaire (no difficulty, mild perceived difficulty, moderate perceived difficulty, or unable to hear at all) and does not represent clinically diagnosed hearing loss. Anxiety severity was assessed using the Generalized Anxiety Disorder-7 (GAD-7) scale. Perceived stress was assessed by self-report and categorized into four levels. Hypertension, diabetes mellitus, and dyslipidemia were defined based on physician diagnosis. Workplace noise exposure was defined as self-reported exposure to workplace noise severe enough to interfere with verbal communication. Daily earphone use duration reflects average listening time in noisy environments. Combined noise exposure categories were defined based on combinations of workplace noise exposure (Yes vs No) and earphone use duration in noisy environments (none, ≤120 min/day, >120 min/day). The category “Unable to hear at all” contained only two unweighted observations (0 with dizziness and 2 without dizziness); weighted estimates are therefore unstable and presented for completeness. Audiometric hearing status was determined using pure-tone average (PTA) thresholds at 0.5, 1, 2, and 4 kHz, applying a both-ear criterion. Participants were classified as having normal hearing (≤25 dB HL, both-ear criterion), mild hearing loss (26–40 dB HL), or moderate-or-worse hearing loss (>40 dB HL). Participants were classified as “not classifiable” if audiometric data were missing or if test conditions did not meet quality standards (e.g., excessive ambient noise in the testing booth). SE, standard error; GAD-7, generalized anxiety disorder-7; dB HL: decibels hearing level.

Participants reporting dizziness had significantly higher levels of perceived stress and anxiety severity. Subjective hearing discomfort and tinnitus severity were more prevalent among those with dizziness; however, objective audiometric hearing loss (PTA > 25 dB HL) did not differ significantly between groups.

### Covariates and multivariable adjustment

In fully adjusted models, female sex was independently associated with higher odds of dizziness. Psychological factors demonstrated the strongest associations: increasing anxiety severity (GAD-7 categories) and higher perceived stress levels were consistently and significantly associated with dizziness across models. In contrast, physician-diagnosed cardiometabolic conditions, including hypertension, diabetes mellitus, and dyslipidemia, showed weaker or non-significant associations after multivariable adjustment.

Adjustment for sociodemographic and psychological factors modestly attenuated the association between workplace noise exposure and dizziness compared with minimally adjusted models, suggesting partial confounding. However, the direction of association remained consistent across exposure specifications. The crude association between workplace noise exposure and dizziness was modestly stronger and became attenuated after adjustment for demographic and psychological factors.

### Associations in the overall population

Under the categorical exposure specification (Table 2), workplace noise exposure showed a directionally positive but non-significant association with dizziness (OR 1.23, 95% CI 0.97–1.56). When earphone duration was modeled categorically, the highest exposure category (>120 min/day) demonstrated elevated odds compared with non-users (OR 1.92, 95% CI 0.93–3.97), although confidence intervals were wide. The intermediate exposure categories were not statistically significant (≤60 min/day: OR 1.19, 95% CI 0.87–1.63; 60–120 min/day: OR 0.96, 95% CI 0.44–2.09). A non-significant linear trend across increasing earphone exposure categories was observed (*p*-for trend = 0.112).

**Table 2:** Associations between noise exposure and dizziness in the overall population.

Exposure specification	Adjusted OR (95% CI)	*p*-value
(1) Categorical exposure specification		
Workplace noise exposure		
No	1.00 (ref)	
Yes	1.23 (0.97–1.56)	0.083
Earphone use in noisy settings (time/day)		
None	1.00 (ref)	
≤60 min/day	1.19 (0.87–1.63)	0.289
61–120 min/day	0.96 (0.44–2.09)	0.914
>120 min/day	1.92 (0.93–3.97)	0.079
*p*-for trend		0.112
(2) Continuous exposure specification		
Workplace noise exposure (no vs. yes)		
No	1.00 (ref)	
Yes	1.24 (0.98–1.56)	0.070
Log-transformed earphone exposure time (ln [min + 1])	1.05 (0.98–1.12)	0.196
(3) Joint exposure specification		
Joint noise exposure categories		
No noise exposure	1.00 (ref)	
Earphone ≤120 min/day only	1.27 (0.93–1.74)	0.129
Earphone >120 min/day only	1.35 (0.56–3.24)	0.501
Workplace noise exposure only	1.27 (0.99–1.63)	0.065
Workplace noise exposure + earphone ≤120 min/day	0.88 (0.38–2.03)	0.770
Workplace noise exposure + earphone >120 min/day	5.03 (1.35–19.05)	0.018

Adjusted odds ratios (ORs) and 95% confidence intervals (CIs) were estimated using survey-weighted logistic regression models. Models were fully adjusted for age, sex, household income, education, smoking status, alcohol consumption, aerobic physical activity, physician-diagnosed hypertension, diabetes mellitus, dyslipidemia, perceived stress, anxiety severity (GAD-7 categories), and tinnitus severity. Joint noise exposure categories were defined as mutually exclusive groups based on combinations of workplace noise exposure (yes vs. no) and earphone use duration (none, ≤120 min/day, >120 min/day). All analyses accounted for the complex survey design of KNHANES. No statistically significant interaction between workplace noise exposure and earphone use was observed (*p*-for interaction = 0.276).

In the continuous exposure specification, workplace noise exposure remained directionally associated with dizziness (OR 1.24, 95% CI 0.98–1.56), while log-transformed earphone exposure time showed a modest but non-significant association (OR 1.05, 95% CI 0.98–1.12).

Under the joint exposure specification, participants with both workplace noise exposure and earphone use >120 min per day showed markedly higher odds of dizziness (OR 5.03, 95% CI 1.35–19.05), whereas single-exposure categories showed more modest associations. No statistically significant interaction between workplace noise exposure and earphone use was observed (*p*-for interaction = 0.276).

### Audiometric normal subgroup

Among participants with normal audiometric hearing (PTA ≤ 25 dB HL), associations were more pronounced (Table 3). In the categorical specification, the highest earphone exposure category (>120 min/day) was significantly associated with dizziness (OR 2.28, 95% CI 1.07–4.83). The linear trend across ordered earphone duration categories remained statistically significant in this subgroup. Similarly, log-transformed earphone exposure time was significantly associated with dizziness (*p* = 0.039). Exploratory shape analyses further showed significance for the raw linear term, but not for hinge-at-120 or quadratic curvature terms, supporting a gradual monotonic increase rather than a discrete threshold at 120 min/day (Supplementary Table S5).

**Table 3:** Associations between noise exposure and dizziness among adults with normal audiometric hearing.

Exposure specification	Adjusted OR (95% CI)	*p*-value
(1) Categorical exposure specification		
Workplace noise exposure		
No	1.00 (ref)	
Yes	1.30 (0.97–1.75)	0.060
Earphone use in noisy settings (time/day)		
None	1.00 (ref)	
≤60 min/day	1.25 (0.89–1.76)	0.206
61–120 min/day	1.35 (0.56–3.24)	0.502
>120 min/day	2.28 (1.07–4.83)	0.032
*p*-for trend		0.008
(2) Continuous exposure specification		
Workplace noise exposure		
No	1.00 (ref)	
Yes	1.31 (0.98–1.76)	0.069
Log-transformed earphone exposure time (ln [min + 1])	1.08 (1.00–1.16)	0.039
(3) Joint exposure specification		
Joint noise exposure categories		
No noise exposure	1.00 (ref)	
Earphone ≤120 min/day only	1.43 (1.04–1.97)	0.027
Earphone >120 min/day only	1.73 (0.70–4.31)	0.239
Workplace noise exposure only	1.37 (1.01–1.86)	0.043
Workplace noise exposure + earphone ≤120 min/day	0.82 (0.27–2.48)	0.723
Workplace noise exposure + earphone >120 min/day	5.14 (1.38–19.14)	0.015

Adjusted odds ratios (ORs) and 95% confidence intervals (CIs) were estimated using survey-weighted logistic regression models. Models were fully adjusted for age, sex, household income, education, smoking status, alcohol consumption, aerobic physical activity, physician-diagnosed hypertension, diabetes mellitus, dyslipidemia, perceived stress, anxiety severity (GAD-7 categories), and tinnitus severity. Joint noise exposure categories were defined as mutually exclusive groups based on combinations of workplace noise exposure (yes vs. no) and earphone use duration (none, ≤120 min/day, >120 min/day). All analyses accounted for the complex survey design of KNHANES. No statistically significant interaction between workplace noise exposure and earphone use was observed (*p*-for interaction = 0.353).

Under the joint exposure specification, participants with both workplace noise exposure and earphone use >120 min per day showed markedly higher odds of dizziness (OR 5.14, 95% CI 1.38–19.14). Formal interaction testing in the full survey-weighted model did not show statistically significant effect modification by hearing status (p for interaction = 0.204). Separately, no statistically significant interaction between workplace noise exposure and earphone use was observed (p for interaction = 0.353).

### Sensitivity analyses

Sensitivity analyses using recurrent dizziness or imbalance (≥2 episodes; DZ3) as the outcome showed a broadly similar pattern of associations in both the overall population and the normal-hearing subgroup (Supplementary Tables S3, S4). In particular, the joint exposure category combining workplace noise exposure and earphone use >120 min/day remained associated with higher odds of recurrent dizziness in both the overall population (OR 7.65, 95% CI 2.00–29.25) and the normal-hearing subgroup (OR 7.92, 95% CI 2.11–29.76).

## Discussion

In this nationally representative sample, associations between individual noise exposure indicators and dizziness were of modest magnitude in the overall population but became more apparent among adults with normal audiometric hearing. This pattern suggests that associations between noise exposure and dizziness-related symptoms may be difficult to detect in heterogeneous populations yet become more discernible when background risk is reduced.

Dizziness is influenced by diverse etiologies, including central vestibular disorders, functional dizziness, metabolic disease, and vascular pathology (4, 29, 30). In the present analysis, psychological factors—particularly anxiety severity and perceived stress—demonstrated stronger associations with dizziness than most noise exposure indicators, underscoring the multifactorial nature of the outcome. In older adults, dizziness is often considered a geriatric syndrome reflecting cumulative multisystem decline (19). Within such heterogeneous contexts, exposure-specific effects may be attenuated or masked.

In addition, while symptom-based classifications of dizziness are commonly used in clinical settings, international efforts have sought to standardize vestibular symptom terminology and establish structured symptom domains (31). However, prior studies have reported substantial overlap and variability in patient-reported symptom descriptions (32, 33), suggesting that the added value of detailed symptom subtyping in population-based research remains uncertain. Dizziness in this study was interpreted as reflecting a broad, nonspecific symptom rather than a discrete clinical entity, consistent with the survey-based operational definitions used in large-scale epidemiologic studies (Supplementary Table S1).

The attenuation of associations may reflect the influence of multiple coexisting factors that dilute exposure-specific effects. In the overall sample, individual noise exposure metrics showed directionally positive but modest associations that were attenuated after adjustment. Although the highest earphone exposure category demonstrated elevated odds, the overall linear trend across ordered earphone duration categories did not reach statistical significance.

However, when analyses were restricted to individuals with normal audiometric thresholds—thereby reducing confounding from age-related sensory impairment (34–36)—the association between noise exposure and dizziness became more apparent and reached statistical significance. This pattern suggests improved detectability of exposure-related associations under reduced background heterogeneity rather than evidence of increased biological susceptibility.

Consistent with this interpretation, formal interaction testing between noise exposure and hearing status in the full survey-weighted model did not show a statistically significant difference in effect size by hearing status.

Although we categorized earphone-use duration using a pragmatic >120 min/day threshold, our exploratory analyses did not support a sharp biological cutoff at that point. In the normal-hearing subgroup, continuous and log-transformed exposure terms were significant, whereas hinge-at-120 and quadratic terms were not, suggesting a gradual exposure-response pattern rather than a distinct threshold. This interpretation aligns with the WHO–ITU framework, which emphasizes cumulative sound dose and recognizes uncertainty in individual susceptibility (24). Thus, the >120 min/day category should be interpreted as a practical exposure-stratification threshold rather than a universal biologic breakpoint. Vulnerability to noise-related inner-ear effects may differ across individuals according to host factors such as pre-existing auditory symptoms, family history, ototoxic exposures, or other biological susceptibilities, although the determinants of such variability remain incompletely understood.

Experimental studies have demonstrated that sound and vibration can activate vestibular receptors (9, 11–14). Noise exposure has also been associated with oxidative stress and microvascular changes within the inner ear (37–39), mechanisms that could plausibly affect both cochlear and vestibular structures. Recent animal models further support the possibility of noise-induced vestibular dysfunction (40).

Importantly, these mechanisms do not require measurable hearing threshold shifts. Cochlear synaptopathy can occur without permanent audiometric loss (6), suggesting that subclinical sensory stress may precede detectable hearing impairment. The stronger associations observed under joint exposure patterns further supports the plausibility that overall acoustic exposure, rather than isolated exposure measures alone, may be relevant. While our cross-sectional data cannot establish such mechanisms, the consistency of associations across categorical, trend-based, and joint exposure specifications supports biological plausibility. These findings were further supported by sensitivity analyses using recurrent dizziness (≥2 episodes), which demonstrated consistent patterns of association in both the overall population and the normal-hearing subgroup (Supplementary Tables S3, S4).

Rather than indicating that noise exposure directly causes dizziness, these findings support a life-course accumulation framework (16). Repeated acoustic exposure across adulthood may incrementally contribute to dizziness-related symptom burden. In populations where other etiologies are prevalent, this contribution may be difficult to detect. However, in relatively lower-background-risk groups—such as middle-aged adults without audiometric hearing loss—the signal becomes more apparent.

This interpretation does not imply that individuals with normal hearing are uniquely vulnerable, nor that dizziness predicts future hearing loss. Instead, it highlights the importance of considering cumulative environmental exposures in the broader trajectory of vestibular health.

While comprehensively quantifying all sources of environmental noise in a general community is methodologically challenging, evaluating multiple domains of exposure—such as integrating occupational noise with recreational earphone use—provides a more accurate proxy for an individual’s total acoustic burden. Consistent with the concept of cumulative noise exposure (41), our results demonstrate that capturing combined exposure across multiple life domains yields greater statistical detectability than analyzing single environmental factors in isolation, as reflected in the markedly elevated odds observed under joint exposure categories compared with individual exposure metrics (Table 2).

Furthermore, extensive experimental and clinical evidence has established that acoustic trauma can induce physical and metabolic damage not only to the cochlea but also to the vestibular end-organs, particularly the saccule (10, 15). Extrapolated to the population level, these established neurotological mechanisms offer one possible explanation for why cumulative acoustic exposure may be associated with a higher prevalence of generalized dizziness or unsteadiness in real-world settings.

Therefore, despite the limitations inherent in a broad survey-based definition of dizziness, the observed associations may reflect an epidemiologic signal linking noise exposure to dizziness, warranting further study in independent cohorts.

Finally, if these findings represent a genuine, underrecognized mechanism linking everyday noise exposure to dizziness—or if alternative pathophysiological pathways are suspected to challenge existing paradigms—it is imperative to verify the reproducibility of this phenomenon. Future epidemiological investigations leveraging independent, large-scale international cohorts, such as the United States National Health and Nutrition Examination Survey (US NHANES) or the United Kingdom Biobank, are warranted to confirm these cross-cultural dynamics and further elucidate the underlying pathophysiology.

Several limitations warrant consideration. First, the cross-sectional design precludes causal inference. Second, noise exposure was self-reported, and actual acoustic dose depends on volume settings, device characteristics, and environmental context (24, 42). Because exposure duration does not directly measure acoustic intensity, non-differential misclassification is likely and would be expected to bias associations toward the null. Third, some variables had missing values due to non-participation in specific examinations; however, these were handled using a complete-case approach and are unlikely to have substantially biased the observed associations. Fourth, dizziness was self-reported and the term “dizziness” alone is not sufficient to establish a direct link between noise exposure and the vestibular system; it may also be associated with presyncope or orthostatic hypotension. In addition, survey-based clinical covariates may not fully capture participants’ clinical status, which may vary according to undiagnosed disease, medication use, disease control, severity, and duration. Finally, objective vestibular testing was not available, and further studies are needed to determine whether the observed association reflects specific vestibular disorders, including BPPV and Ménière disease.

In conclusion, while the associations between individual noise metrics and dizziness were modest in the overall population, our findings highlight the epidemiological significance of cumulative acoustic exposure. The pronounced associations observed under joint exposure patterns—particularly among individuals with normal audiometric hearing—suggest that repeated acoustic stress across occupational and recreational contexts may be associated with dizziness-related symptoms. These findings warrant cautious interpretation and underscore the need for longitudinal studies incorporating objective noise dosimetry and detailed vestibular assessments to clarify temporality and determine whether this association extends to specific vestibular disorders.

## Data Availability

Publicly available datasets were analyzed in this study. The data are available from the Korea National Health and Nutrition Examination Survey (KNHANES): https://knhanes.kdca.go.kr.
