# Supplementary material for: Noise exposure and dizziness in middle-aged adults: a nationally representative study
Source: Front Neurol. 2026 May 19;17:1818481. doi: 10.3389/fneur.2026.1818481 (PMC13225996; doi:10.3389/fneur.2026.1818481)
Supplement: Supplementary file 1 [file Data_Sheet_1.pdf]

Supplementary Table S1. Dizziness-related questionnaire items used in the study (KNHANES)

Item	English translation
DZ2	Experience of falling or collapsing due to loss of balance without an external cause
DZ3	Recurrent dizziness or imbalance (≥2 episodes)
DZ4	Difficulty maintaining balance while standing
DZ5	Difficulty walking due to imbalance
DZ6	Chronic or persistent dizziness
DZ7	Recurrent falls

Supplementary Table S2. Distribution of dizziness-related questionnaire items

Variable	Yes, n (weighted %)	No, n (weighted %)
DZ2 (fall without external cause)	42 (1.2%)	1030 (30.5%)
DZ3 (recurrent dizziness ≥2 episodes)	805 (23.8%)	267 (7.9%)
DZ4 (postural instability)	17 (0.5%)	1055 (31.3%)
DZ5 (gait imbalance)	15 (0.4%)	1057 (31.3%)
DZ6 (chronic dizziness)	91 (2.7%)	981 (29.1%)
DZ7 (recurrent falls)	1 (<0.1%)	1071 (31.7%)

These items were assessed using a skip pattern among participants who reported dizziness. Therefore, a substantial proportion of participants were classified as “not applicable,” and percentages may not sum to 100%.

Supplementary Table S3. Sensitivity analysis using recurrent dizziness (≥2 episodes) as the outcome in the overall population

Exposure	OR (95% CI)
<120 min (non-occupational)	1.37 (0.99–1.91)
>120 min (non-occupational)	1.11 (0.38–3.27)
Occupational only	1.23 (0.93–1.63)
Earphone ≤120 min/day + occupational noise	0.79 (0.32–1.96)
Earphone >120 min/day + occupational noise	7.65 (2.00–29.25)

Models were adjusted for age, sex, household income, aerobic physical activity, tinnitus severity, perceived stress, anxiety (GAD-7), alcohol consumption, smoking status, and tinnitus duration using a complex survey design.

Supplementary Table S4. Sensitivity analysis using recurrent dizziness (≥2 episodes) among participants with normal hearing

Exposure	OR (95% CI)
<120 min (non-occupational)	1.54 (1.09–2.19)
>120 min (non-occupational)	1.39 (0.46–4.24)
Occupational only	1.35 (0.98–1.86)
Earphone ≤120 min/day + occupational noise	0.81 (0.24–2.71)
Earphone >120 min/day + occupational noise	7.92 (2.11–29.76)

Models were adjusted for age, sex, household income, aerobic physical activity, tinnitus severity, perceived stress, anxiety (GAD-7), alcohol consumption, smoking status, and tinnitus duration using a complex survey design.

Supplementary Table S5. Exploratory continuous-shape analyses of earphone-use duration and dizziness in the overall population and the audiometric normal-hearing subgroup

Population	Model	Estimate	P value
Overall population	Raw linear	OR 1.002 (0.999–1.005)	0.166
	Log-transformed	OR 1.05 (0.98–1.12)	0.196
	Hinge-at-120	OR 1.003 (0.993–1.013)	0.521
	Quadratic	B = -3.539E-7	0.972
Normal-hearing subgroup	Raw linear	OR 1.003 (1.000–1.006)	0.034
	Log-transformed	OR 1.08 (1.00–1.16)	0.039
	Hinge-at-120	OR 1.002 (0.989–1.015)	0.791
	Quadratic	B = -3.196E-6	0.799

Adjusted odds ratios (ORs) and 95% confidence intervals (CIs) were estimated using survey-weighted logistic regression models with the same fully adjusted covariate set as in the main analyses. For the hinge model, the value shown corresponds to the post-120-minute hinge term only. For the quadratic model, the squared term ($C120^2$) is presented using the regression coefficient (B) rather than OR because exponentiated estimates for polynomial terms are difficult to interpret and may appear artificially rounded to 1.000. Log-transformed estimates were rounded to match the corresponding values presented in the main Tables 2 and 3.
